# Association of Prothrombotic and Oxidative Stress Biomarkers With the Risk of Microvascular Dysfunction in Patients With Type 2 Diabetes Mellitus

**DOI:** 10.7759/cureus.110875

**Published:** 2026-06-15

**Authors:** Muhammad Omair Zahid, Talat Bashir, Lamia Rafique, Iffat Ara Aziz, Iman Zishan, Husnain Hashim

**Affiliations:** 1 Cardiology, Rawalpindi Institute of Cardiology, Rawalpindi, PAK; 2 Medicine, Maryam Medicare Hospital, Vehari, PAK; 3 Cardiology, Tbilisi State Medical University, Tbilisi, GEO; 4 Biochemistry, Baqai Medical University, Karachi, PAK; 5 Obstetrics and Gynaecology, Tbilisi State Medical University, Tbilisi, GEO; 6 Neurology, Fauji Foundation Hospital, Rawalpindi, PAK

**Keywords:** endothelial dysfunction, microvascular dysfunction, oxidative stress, pro-thrombotic biomarker, type 2 diabetes mellitus

## Abstract

Background

Type 2 diabetes mellitus is a common metabolic disorder associated with progressive vascular complications. Microvascular dysfunction is an important cause of diabetic nephropathy, retinopathy, and neuropathy. Oxidative stress and prothrombotic activity are considered major contributors to vascular injury among diabetic patients. This study aimed to evaluate the association of prothrombotic and oxidative stress biomarkers with microvascular dysfunction risk in patients with type 2 diabetes mellitus.

Methods

This hospital-based observational cross-sectional study was conducted at Maryam Medicare Hospital, Vehari, Pakistan, from February 2025 to January 2026. A total of 200 patients with type 2 diabetes mellitus were included using consecutive sampling. Patients were divided into low-risk and high-risk microvascular dysfunction groups based on clinical and biochemical assessment. Prothrombotic biomarkers including fibrinogen, D-dimer, and plasminogen activator inhibitor-1 (PAI-1) were measured along with oxidative stress biomarkers including malondialdehyde (MDA), total antioxidant capacity (TAC), superoxide dismutase (SOD), glutathione (GSH), and catalase activity. Statistical analysis was performed using IBM SPSS Statistics for Windows, Version 26 (Released 2018; IBM Corp., Armonk, New York, United States).

Results

Patients with high microvascular dysfunction risk showed significantly increased fibrinogen (421.5 ± 72.4 vs 312.8 ± 48.6 mg/dL), D-dimer (458.7 ± 101.5 vs 228.4 ± 62.1 ng/mL), PAI-1 (34.2 ± 8.7 vs 18.7 ± 5.4 ng/mL), and MDA levels (4.86 ± 0.92 vs 2.41 ± 0.58 nmol/mL) compared with the low-risk group (p < 0.001). Antioxidant biomarkers including TAC, SOD, GSH, and catalase were significantly reduced in high-risk patients (p < 0.001). MDA showed the highest predictive value for microvascular dysfunction risk with an area under the curve (AUC) of 0.871.

Conclusion

Elevated thrombotic and oxidative stress biomarkers were significantly associated with the presence of microvascular complications in patients with type 2 diabetes mellitus. These findings suggest that these biomarkers may reflect the burden of vascular injury and oxidative stress in patients with established diabetic microvascular complications.

## Introduction

Type 2 diabetes mellitus is one of the most common chronic metabolic diseases worldwide, and its prevalence is rapidly increasing in developing countries, including Pakistan [1]. Persistent hyperglycemia in diabetes causes long-term damage to blood vessels and different organs of the body [2]. Among diabetic complications, microvascular dysfunction is particularly important because it leads to diabetic nephropathy, retinopathy, and neuropathy, which significantly increase morbidity and reduce quality of life [3]. Early identification of patients at higher risk of microvascular complications is important for better disease management and prevention of irreversible vascular damage [4].

Microvascular dysfunction in diabetes develops through multiple pathological mechanisms including chronic inflammation, endothelial dysfunction, oxidative stress, and activation of coagulation pathways [5,4]. Persistent elevation of blood glucose increases production of reactive oxygen species (ROS), which disturb the balance between oxidants and antioxidants inside the body [6,7]. Excessive oxidative stress damages lipids, proteins, and vascular endothelial cells, leading to impaired microcirculation and tissue injury [8]. Malondialdehyde (MDA) is a commonly used marker of lipid peroxidation and reflects oxidative damage caused by free radicals. In contrast, antioxidant defense systems including superoxide dismutase (SOD), glutathione (GSH), catalase, and total antioxidant capacity (TAC) help protect tissues against oxidative injury [9]. Reduced antioxidant activity has been reported in diabetic patients with vascular complications [10].

In addition to oxidative stress, diabetes is also associated with a prothrombotic state. Endothelial dysfunction, platelet activation, increased blood viscosity, and impaired fibrinolysis contribute to abnormal coagulation activity in diabetic patients [11]. Fibrinogen is an important coagulation protein that increases blood clot formation and vascular inflammation [12]. D-dimer reflects activation of fibrin turnover and coagulation pathways, while plasminogen activator inhibitor-1 (PAI-1) inhibits fibrinolysis and promotes persistence of thrombi. Elevated levels of these biomarkers may contribute to impaired blood flow and progression of diabetic microvascular injury [13].

The present study was designed to evaluate the association of prothrombotic biomarkers, including fibrinogen, D-dimer, and PAI-1, along with oxidative stress biomarkers including MDA, TAC, SOD, GSH, and catalase activity, with microvascular dysfunction risk in patients with type 2 diabetes mellitus.

## Materials and methods

This hospital-based observational cross-sectional study was conducted at the Department of Medicine and Clinical Laboratory Services, Maryam Medicare Hospital, Vehari, Pakistan, over a period of 12 months starting on 1 February 2025. The study was designed and reported in accordance with the Strengthening the Reporting of Observational Studies in Epidemiology (STROBE) guidelines for observational studies. Ethical approval was obtained from the Ethical Review Board of Maryam Medicare Hospital, Vehari, under Ref No. ERB/MMH/25-234, dated January 7, 2025. Written informed consent was obtained from all participants prior to enrollment, and confidentiality of patient information was maintained throughout the study.

The study included adult patients of either gender aged between 35 and 70 years with previously diagnosed type 2 diabetes mellitus attending outpatient and inpatient medical services of the hospital. Patients with a duration of diabetes greater than one year and stable anti-diabetic treatment for at least three months were included to minimize acute metabolic fluctuations. Patients with known macrovascular complications, including myocardial infarction, stroke, peripheral arterial disease, severe heart failure, chronic liver disease, malignancy, autoimmune disorders, acute infections, chronic inflammatory conditions, pregnancy, smoking history, alcohol use, or use of antioxidant supplements, corticosteroids, anticoagulants, or anti-inflammatory drugs within the previous three months were excluded because these conditions may independently influence oxidative stress and thrombotic biomarkers. Patients with renal failure (estimated glomerular filtration rate (eGFR) <30 mL/min/1.73 m^2^) were also excluded due to altered oxidative and coagulation profiles.

The sample size was calculated using OpenEpi version 3.01 (Dean AG, Sullivan KM, Soe MM. OpenEpi: Open Source Epidemiologic Statistics for Public Health) for analytical cross-sectional studies [14] by assuming a prevalence of microvascular dysfunction risk among type 2 diabetic patients of 50% [15], confidence interval of 95%, margin of error of 5%, and study power of 80%, resulting in a minimum required sample size of 196 participants. To compensate for possible incomplete data or sample loss, 10% additional recruitment was planned, and a final target sample size of approximately 215 participants was considered adequate. A non-probability consecutive sampling technique was employed, and all eligible participants presenting during the study period were invited to participate until the required sample size was achieved.

Detailed demographic and clinical information, including age, gender, body mass index, duration of diabetes, blood pressure, medication history, fasting blood glucose, and glycated hemoglobin levels, were collected through participant interviews and review of patients' medical records. To minimize confounding, patients with conditions known to affect thrombosis or oxidative stress were excluded, while age, gender, body mass index, duration of diabetes, glycemic control, hypertension, and dyslipidemia were additionally considered during statistical adjustment. Participants were instructed to observe overnight fasting for 10-12 hours before blood collection. Approximately 8-10 mL of venous blood was collected under aseptic conditions. Serum and plasma were separated by centrifugation at 3000 rpm for 10 minutes and stored at -80°C until biochemical analysis.

Assessment of diabetic microvascular complications was based on documented clinical and laboratory findings obtained from medical records and physician evaluations. Diabetic nephropathy was identified by the presence of albuminuria as determined by urinary albumin-to-creatinine ratio measurements. Diabetic neuropathy was identified through documented clinical assessment and physician diagnosis recorded in the medical records. Diabetic retinopathy was identified from ophthalmologic examination findings available in patient records. Participants with one or more documented diabetic microvascular complications were assigned to the microvascular complications group (case group), while patients without evidence of diabetic microvascular complications were classified as the no microvascular complications group (control group). The presence of any single complication was sufficient for group assignment, and no weighting system was applied.

Oxidative stress biomarkers including MDA, TAC, SOD, GSH, and catalase activity were estimated using standard spectrophotometric methods. Serum MDA levels were determined by thiobarbituric acid reactive substance (TBARS) assay according to the method of Ohkawa et al. [16] using spectrophotometric detection at 532 nm. TAC was measured by following the method described by Nisar et al. [7]. SOD activity was estimated by inhibition of the nitroblue tetrazolium reduction method as described by Misra and Fridovich [17]. Catalase activity was determined by hydrogen peroxide decomposition assay according to the method of Hadwan [18], while reduced GSH levels were measured using Ellman’s reagent method [19]. Fasting blood glucose was analyzed by glucose oxidase-peroxidase (GOD-POD) method using commercially available diagnostic kits manufactured by Randox Laboratories (Randox Laboratories Ltd., Crumlin, County Antrim, United Kingdom), whereas glycated hemoglobin (HbA1c) was measured by high-performance liquid chromatography-based automated analysis using Bio-Rad D-10 analyzer (Bio-Rad Laboratories, Hercules, CA, USA). Prothrombotic biomarkers including PAI-1 were quantified using commercially available sandwich ELISA kits (Invitrogen Human PAI-1 ELISA Kit, Cat No. BMS2033, Thermo Fisher Scientific, USA) according to manufacturer instructions. Fibrinogen and D-dimer levels were analyzed using automated coagulation analyzer-based methods with commercially available reagent kits.

All laboratory analyses were performed in duplicate to improve analytical reliability, and internal quality control sera were run with each batch of samples. Data entry and verification were performed independently by two researchers to reduce transcription errors. Missing data exceeding 10% for any participant were excluded from final analysis, while isolated missing laboratory values were handled using pairwise deletion during statistical analysis to preserve sample integrity without introducing artificial imputation bias.

Statistical analysis was carried out using IBM SPSS Statistics for Windows, Version 26 (Released 2018; IBM Corp., Armonk, New York, United States). Continuous variables were tested for normality using the Shapiro-Wilk test. Normally distributed data were expressed as mean ± standard deviation, whereas non-normally distributed variables were presented as median and interquartile range. Independent-samples t-test or Mann-Whitney U test was used for comparison between groups as appropriate. Categorical variables were analyzed using chi-square or Fisher’s exact test. Pearson or Spearman correlation analysis was performed to determine associations between oxidative stress biomarkers, prothrombotic markers, and indicators of microvascular dysfunction. Multivariable logistic regression analysis was conducted to identify independent predictors of high microvascular dysfunction risk after adjustment for potential confounders. A p-value of less than 0.05 was considered statistically significant.

## Results

A total of 215 eligible patients with type 2 diabetes mellitus were enrolled. Eleven participants were excluded because of incomplete laboratory data, and four withdrew consent before completion of the study. Consequently, 200 participants were included in the final statistical analysis, exceeding the minimum required sample size of 196. Among them, 118 (59%) patients were categorized as prevalent microvascular complications (case group), while 82 (41%) patients were classified as patients without microvascular complications (control group) based on clinical and biochemical indicators of diabetic microvascular complications.

The demographic and baseline clinical characteristics of study participants are summarized in Table 1. Patients in the case group had significantly higher age, longer duration of diabetes, fasting blood glucose, HbA1c, systolic blood pressure, and body mass index compared with the low-risk group (p < 0.05).

**Table 1:** Demographic and Clinical Characteristics of Study Participants Data are presented as mean ± standard deviation or number (percentage). An independent-samples t-test was used for comparison of continuous variables, while a chi-square test was used for categorical variables. Chi-square statistics are presented with degrees of freedom and the Phi coefficient as an effect size measure. A p-value <0.05 was considered statistically significant.

Variable	Patients Without Microvascular Complications (Control Group) (N = 82)	Patients With Prevalent Microvascular Complications (Case Group) (N = 118)	Test Statistic	p-value
Age (years)	51.8 ± 7.2	58.6 ± 8.1	t(198) = 6.08	<0.001
Male/Female (n)	39/43	61/57	χ^2^(1) = 0.50, Phi = 0.05	0.481
Duration of Diabetes (years)	6.2 ± 2.8	11.1 ± 4.5	t(198) = 9.02	<0.001
Body Mass Index (kg/m^2^)	26.4 ± 3.1	29.2 ± 4.2	t(198) = 5.24	<0.001
Systolic Blood Pressure (mmHg)	126.5 ± 11.8	139.7 ± 14.2	t(198) = 7.12	<0.001
Diastolic Blood Pressure (mmHg)	79.2 ± 7.6	84.1 ± 8.3	t(198) = 4.29	0.001
Fasting Blood Glucose (mg/dL)	142.6 ± 25.8	189.5 ± 38.4	t(198) = 9.87	<0.001
HbA1c (%)	7.1 ± 0.8	9.0 ± 1.3	t(198) = 11.84	<0.001
Hypertension, n (%)	28 (34.1)	69 (58.5)	χ^2^(1) = 11.62, Phi = 0.24	0.001
Dyslipidemia, n (%)	24 (29.3)	63 (53.4)	χ^2^(1) = 11.31, Phi = 0.23	0.001

The comparison of prothrombotic biomarkers between study groups demonstrated significantly elevated levels of fibrinogen, D-dimer, and PAI-1 in patients with prevalent microvascular dysfunction compared to control group patients (Table 2). Independent-samples t-test showed statistically significant differences for all measured thrombotic biomarkers (p < 0.001).

**Table 2:** Comparison of Prothrombotic Biomarkers Between Study Groups Data are expressed as mean ± standard deviation. An independent-samples t-test was used to compare group means. A p-value <0.05 was considered statistically significant. PAI-1: plasminogen activator inhibitor-1, ng/mL: nanogram per milliliter, mg/dL: milligram per deciliter, SD: standard deviation

Biomarker	Reference Range	Patients Without Microvascular Complications (Control Group; N = 82)	Patients With Prevalent Microvascular Complications (Case Group; N = 118)	p-value
Fibrinogen (mg/dL)	200-400	312.8 ± 48.6	421.5 ± 72.4	<0.001
D-dimer (ng/mL)	<500	228.4 ± 62.1	458.7 ± 101.5	<0.001
PAI-1 (ng/mL)	4-43	18.7 ± 5.4	34.2 ± 8.7	<0.001

Oxidative stress biomarkers showed marked alterations in the case group. Serum MDA levels were significantly elevated, while TAC, SOD, GSH, and catalase activities were significantly reduced in patients with increased microvascular dysfunction risk (Table 3).

**Table 3:** Comparison of Oxidative Stress Biomarkers Between Study Groups Data are shown as mean ± standard deviation. An independent-samples t-test was used for intergroup comparison. A p-value <0.05 was considered statistically significant. MDA: malondialdehyde, TAC: total antioxidant capacity, SOD: superoxide dismutase, GSH: reduced glutathione, U/mL: units per milliliter, nmol/mL: nanomole per milliliter, µmol/L: micromole per liter

Biomarker	Reference Range	Patients Without Microvascular Complications (Control Group; N = 82)	Patients With Prevalent Microvascular Complications (Case Group; N = 118)	p-value
MDA (nmol/mL)	1.0-3.5	2.41 ± 0.58	4.86 ± 0.92	<0.001
TAC (mmol Trolox Eq/L)	1.3-2.0	1.62 ± 0.27	0.94 ± 0.21	<0.001
SOD (U/mL)	6-12	8.72 ± 1.34	5.18 ± 1.09	<0.001
GSH (µmol/L)	30-60	41.5 ± 6.8	25.2 ± 5.1	<0.001
Catalase (U/mL)	50-100	72.6 ± 10.5	48.4 ± 8.7	<0.001

Correlation analysis revealed significant positive associations between MDA and HbA1c, fibrinogen, D-dimer, and PAI-1. In contrast, antioxidant biomarkers including TAC, SOD, GSH, and catalase demonstrated significant negative correlations with HbA1c and thrombotic biomarkers (fibrinogen, D-dimer, and PAI-1). All observed correlations were statistically significant (p < 0.001). Pearson correlation coefficients are presented in Table 4.

**Table 4:** Correlation of Oxidative Stress and Prothrombotic Biomarkers With HbA1c and Microvascular Dysfunction Score Pearson correlation test was applied for normally distributed variables, and Spearman correlation was used where appropriate. Correlation coefficient (r) and p-values are reported. A p-value <0.05 was considered statistically significant. r: correlation coefficient, HbA1c: glycated hemoglobin, MDA: malondialdehyde, TAC: total antioxidant capacity, SOD: superoxide dismutase, GSH: reduced glutathione, PAI-1: plasminogen activator inhibitor-1

Variable	HbA1c	Fibrinogen	D-dimer	PAI-1
MDA	r = 0.612, p < 0.001	r = 0.584, p < 0.001	r = 0.601, p < 0.001	r = 0.548, p < 0.001
TAC	r = -0.528, p < 0.001	r = -0.472, p < 0.001	r = -0.438, p < 0.001	r = -0.421, p < 0.001
SOD	r = -0.493, p < 0.001	r = -0.405, p < 0.001	r = -0.452, p < 0.001	r = -0.387, p < 0.001
GSH	r = -0.571, p < 0.001	r = -0.501, p < 0.001	r = -0.479, p < 0.001	r = -0.442, p < 0.001
Catalase	r = -0.462, p < 0.001	r = -0.416, p < 0.001	r = -0.389, p < 0.001	r = -0.371, p < 0.001

Multivariable logistic regression analysis was performed using simultaneous entry of selected clinical and biomarker variables. Elevated HbA1c, duration of diabetes, fibrinogen, D-dimer, PAI-1, and MDA were independently associated with prevalent microvascular complications, whereas higher TAC and GSH levels demonstrated inverse associations (Table 5). No stepwise variable selection was used.

**Table 5:** Multivariable Logistic Regression Analysis for Predictors of High Microvascular Dysfunction Risk Multivariable binary logistic regression analysis was applied. Adjusted odds ratios (AORs) with 95% confidence intervals (CIs) are reported. The model included adjustment for duration of diabetes and HbA1c. A p-value <0.05 was considered statistically significant. HbA1c: glycated hemoglobin, MDA: malondialdehyde, TAC: total antioxidant capacity, GSH: reduced glutathione, PAI-1: plasminogen activator inhibitor-1

Variable	Adjusted Odds Ratio (AOR)	95% Confidence Interval	p-value
HbA1c (%)	1.84	1.29-2.63	0.001
Duration of Diabetes (years)	1.17	1.06-1.30	0.003
Fibrinogen (per 10 mg/dL Increase)	1.11	1.05-1.18	<0.001
D-dimer (per 50 ng/mL Increase)	1.24	1.10-1.41	<0.001
PAI-1 (per 1 ng/mL Increase)	1.09	1.03-1.16	0.004
MDA (per 1 nmol/mL Increase)	2.68	1.72-4.19	<0.001
TAC (per 0.1 mmol/L Increase)	0.81	0.71-0.92	0.002
GSH (per 1 µmol/L Increase)	0.89	0.84-0.95	<0.001

Receiver operating characteristic (ROC) analysis demonstrated that MDA had the highest diagnostic performance for predicting high microvascular dysfunction risk with an area under the curve of 0.871, followed by D-dimer and fibrinogen (Table 6).

**Table 6:** Receiver Operating Characteristic (ROC) Analysis of Significant Biomarkers for Predicting High Microvascular Dysfunction Risk ROC curve analysis with calculation of AUC. AUC, sensitivity, specificity, and p-values are presented. A p-value <0.05 was considered statistically significant. CI: confidence interval, MDA: malondialdehyde, TAC: total antioxidant capacity, PAI-1: plasminogen activator inhibitor-1

Biomarker	Area Under the Curve (AUC)	95% CI	Sensitivity (%)	Specificity (%)	p-value
MDA	0.871	0.819-0.923	82.2	79.3	<0.001
D-dimer	0.842	0.786-0.897	79.6	75.6	<0.001
Fibrinogen	0.816	0.754-0.879	74.5	73.2	<0.001
TAC	0.804	0.744-0.864	76.1	70.7	<0.001
PAI-1	0.791	0.726-0.856	70.3	71.9	<0.001

## Discussion

Type 2 diabetes mellitus is a major metabolic disorder and an important cause of microvascular complications worldwide [1]. Persistent hyperglycemia damages small blood vessels and leads to endothelial dysfunction, oxidative stress, inflammation, and activation of prothrombotic pathways [2]. In the present study, patients with high microvascular dysfunction risk showed significantly higher oxidative stress and prothrombotic biomarkers compared with low-risk diabetic patients. These findings suggest that oxidative stress and abnormal coagulation activity may play an important role in the progression of diabetic microvascular injury.

In our study, patients with high microvascular dysfunction risk had significantly higher age, longer duration of diabetes, higher fasting glucose, HbA1c, body mass index, and blood pressure levels. These findings are expected because prolonged hyperglycemia gradually damages vascular endothelium and increases oxidative burden. Previous studies also reported that poor glycemic control and longer disease duration are strongly associated with diabetic microvascular complications [3]. Chronic hyperglycemia increases production of ROS, advanced glycation end products, and inflammatory mediators, which finally impair vascular function [4].

The present study demonstrated significantly elevated fibrinogen, D-dimer, and PAI-1 levels in patients with high microvascular dysfunction risk. Diabetes is known to produce a prothrombotic state due to endothelial injury, platelet activation, impaired fibrinolysis, and coagulation abnormalities [11]. Fibrinogen is an acute phase reactant and contributes to blood viscosity and clot formation [20]. Elevated D-dimer reflects increased fibrin turnover and ongoing coagulation activation, while increased PAI-1 inhibits fibrinolysis and promotes persistence of thrombi [21]. Similar findings were reported in previous studies showing increased thrombotic biomarkers in diabetic patients with vascular complications [22].

Our findings also showed significantly higher MDA levels and lower TAC, SOD, GSH, and catalase activity in the high-risk group. MDA is a well-known marker of lipid peroxidation and reflects oxidative damage to cell membranes. Increased MDA in diabetic patients indicates excessive free radical production caused by chronic hyperglycemia [23]. In contrast, antioxidant defenses including SOD, catalase, GSH, and TAC were significantly reduced, suggesting exhaustion of endogenous antioxidant systems due to continuous oxidative stress. Previous studies similarly demonstrated increased oxidative damage and reduced antioxidant enzyme activity in diabetic patients with microvascular complications [24].

The correlation analysis in our study demonstrated positive associations between MDA and prothrombotic biomarkers, while antioxidant parameters showed negative correlations with HbA1c and microvascular dysfunction score. These findings indicate a close interaction between oxidative stress and coagulation abnormalities in diabetes. Oxidative stress damages endothelial cells and decreases nitric oxide bioavailability, leading to endothelial dysfunction and vascular inflammation [25]. This damaged endothelium promotes platelet aggregation and activation of coagulation pathways. Reduced antioxidant activity further worsens vascular injury and promotes progression of microvascular dysfunction. Similar mechanisms have been explained in earlier studies discussing endothelial dysfunction and oxidative stress in diabetes mellitus [26].

Multivariable logistic regression analysis identified HbA1c, fibrinogen, D-dimer, PAI-1, and MDA as independent predictors of high microvascular dysfunction risk, while TAC and GSH showed protective effects. These findings suggest that oxidative and thrombotic biomarkers may independently contribute to vascular injury beyond traditional risk factors. Among all biomarkers, MDA showed the highest diagnostic performance in the ROC analysis. This may be because lipid peroxidation is directly related to endothelial cell damage and reflects overall oxidative burden in diabetic patients [26]. Similar studies also suggested that oxidative stress biomarkers may have potential value for early prediction of diabetic vascular complications [3].

The clinical importance of this study is that combined assessment of oxidative stress and prothrombotic biomarkers may help identify diabetic patients at higher risk of developing microvascular complications before severe clinical damage occurs. The observed associations indicate that thrombotic and oxidative stress biomarkers are closely linked with the presence of diabetic microvascular complications. However, because of the cross-sectional design, the temporal relationship between biomarker alterations and complication development cannot be determined. From a practical perspective, fibrinogen and D-dimer assays are widely available, standardized, and routinely performed in most clinical laboratories, making them potentially more feasible for clinical application. In contrast, biomarkers such as PAI-1, MDA, TAC, and GSH often require specialized assays and are not routinely available in many healthcare settings. Further studies are needed to determine the cost-effectiveness, standardization, and incremental clinical utility of these biomarkers before their incorporation into routine diabetic risk assessment.

Some limitations of the present study should be considered. This was a single-center cross-sectional study with a relatively limited sample size; therefore, causal and temporal relationships could not be established, and reverse causation cannot be excluded. Elevated thrombotic biomarkers may partly reflect established microvascular disease, inflammation, or renal dysfunction rather than preceding these complications. Inflammatory markers such as CRP and detailed eGFR subgroup analyses were not available, which may have resulted in residual confounding. Longitudinal multicenter studies with larger populations are needed to validate these findings and determine the prognostic value of these biomarkers over time.

## Conclusions

Patients with type 2 diabetes mellitus and prevalent microvascular complications showed significantly increased oxidative stress and prothrombotic activity along with impaired antioxidant defense mechanisms. Elevated levels of MDA, fibrinogen, D-dimer, and PAI-1 were significantly associated with the presence of microvascular complications, whereas antioxidant biomarkers demonstrated inverse associations. These findings suggest that thrombotic and oxidative stress biomarkers reflect the vascular and oxidative stress burden in patients with established diabetic microvascular complications. Due to the cross-sectional nature of the study, causal and temporal relationships cannot be determined. Prospective longitudinal studies are warranted to evaluate whether these biomarkers have predictive value for the future development of diabetic microvascular complications.
